# On the Advent of Super-Resolution Microscopy in the Realm of Polycomb Proteins

**DOI:** 10.3390/biology12030374

**Published:** 2023-02-26

**Authors:** Irene Nepita, Simonluca Piazza, Martina Ruglioni, Sofia Cristiani, Emanuele Bosurgi, Tiziano Salvadori, Giuseppe Vicidomini, Alberto Diaspro, Marco Castello, Andrea Cerase, Paolo Bianchini, Barbara Storti, Ranieri Bizzarri

**Affiliations:** 1 Nanoscopy, Istituto Italiano di Tecnologia, Via E. Melen 83, 16152 Genova, Italy; 2 Molecular Microscopy and Spectroscopy, Istituto Italiano di Tecnologia, Via E. Melen 83, 16152 Genova, Italy; 3 R&D Department, Genoa Instruments s.r.l., Via E. Melen 83, 16152 Genova, Italy; 4 Department of Surgical, Medical and Molecular Pathology, and Critical Care Medicine, University of Pisa, Via Roma 65, 56126 Pisa, Italy; 5 DIFILAB, Dipartimento di Fisica, Università degli Studi di Genova, Via Dodecaneso 33, 16146 Genova, Italy; 6 Unit of Cell and Developmental Biology, Department of Biology, University of Pisa, Strada Statale dell’Abetone Brennero 4, 56123 Pisa, Italy; 7 NEST, Scuola Normale Superiore and Istituto Nanoscienze-CNR, Piazza San Silvestro 12, 56127 Pisa, Italy

**Keywords:** chromatin organization, polycomb proteins, PRC1, PRC2, Xist RNA, super-resolution microscopy, STORM, 3D-SIM, oligopaint, FISH

## Abstract

**Simple Summary:**

The genomes of metazoans are organized at multiple spatial scales, ranging from the double helix of DNA to whole chromosomes. The intermediate genomic scale of kilobases to megabases, which corresponds to the 50–300 nm spatial scale, is particularly interesting because the tridimensional arrangement of chromatin is implicated in multiple regulatory mechanisms. Indeed, a crucial hallmark of cellular life is the widespread ordering of many biological processes in nano-/mesoscopic domains (10–200 nm), which now may be revealed by an imaging toolbox referred to as super-resolution microscopy. In this context, polycomb proteins stand as major epigenetic modulators of chromatin function, acting prevalently as repressors of gene transcription. This work reviews the current state-of-the-art super-resolution microscopy applied to polycomb proteins. Of note, super-resolution data have complemented cutting-edge molecular biology methods in providing a rational framework for understanding how polycomb proteins may shape 3D chromatin topologies and functions.

**Abstract:**

The genomes of metazoans are organized at multiple spatial scales, ranging from the double helix of DNA to whole chromosomes. The intermediate genomic scale of kilobases to megabases, which corresponds to the 50–300 nm spatial scale, is particularly interesting, as the 3D arrangement of chromatin is implicated in multiple regulatory mechanisms. In this context, polycomb group (PcG) proteins stand as major epigenetic modulators of chromatin function, acting prevalently as repressors of gene transcription by combining chemical modifications of target histones with physical crosslinking of distal genomic regions and phase separation. The recent development of super-resolution microscopy (SRM) has strongly contributed to improving our comprehension of several aspects of nano-/mesoscale (10–200 nm) chromatin domains. Here, we review the current state-of-the-art SRM applied to PcG proteins, showing that the application of SRM to PcG activity and organization is still quite limited and mainly focused on the 3D assembly of PcG-controlled genomic loci. In this context, SRM approaches have mostly been applied to multilabel fluorescence in situ hybridization (FISH). However, SRM data have complemented the maps obtained from chromosome capture experiments and have opened a new window to observe how 3D chromatin topology is modulated by PcGs.

## 1. Introduction

**Figure 1:**
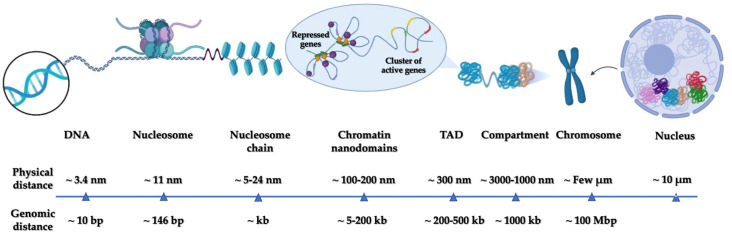
The different scales of chromatin organization, from DNA up to chromosome regions, in the nucleus.

The human genome has the potential to generate hundreds of cell types through a vast repertoire of gene expression patterns. This is shown by the low percentage (1.5%) of the genome allocated to encoding proteins [1], whilst the remaining 98.5% codes for regulatory elements, the functions of most of which are still largely unknown. The approximately 6 billion bases of DNA are wrapped around 30 million nucleosome octamers, forming a complex macromolecular environment named chromatin. Chromatin organization regulates the landscape in which the activity state of the genome is modulated and perpetuated. Two mechanisms serve to increase the information content of the genome, thereby constituting the *epigenome*: chemical modifications of DNA and histones and changes in the local compaction state and nuclear localization of chromatin. These mechanisms are deeply interleaved [2], and the result is the hierarchical organization of chromatin across multiple scales shaped by chromatin-acting proteins (Figure 1) [3]. Starting from the micron scale, individual chromosomes in interphase segregate into chromosome territories. Each chromosome is further subdivided into A and B compartments, which refer to gene-active and gene-inactive regions of a few megabases (Mb) and span several hundredths of nanometers [3]. Below the compartment scale, chromatin organizes into Topological Associated Domains (TADs), which represent dynamic regions of extended interactions between DNA chains [4]. These regions are often insulated from each other by loops pinched by CCCTC-binding factors (CTCFs) and cohesin [3]. TADs comprise DNA segments ranging in size from 100 kilobases (kb) up to several megabases and they are supposed to segregate genes and their distal regulatory elements into compartments crucial for tailored gene regulation [1]. Recently, it was shown that TADs can be further subdivided into smaller chromatin nanodomains (CNDs), the sizes of which are around 100 nm and which embed about 10–100 kb. CNDs are the domains where most enhancer–promoter (E–P) contacts take place [5]. At the lowest hierarchy level, nucleosomes are organized into clutches containing about 1–2 kb of DNA [5].

This complex landscape organization is governed by the intertwined *epigenetic* actions of several moieties, some of which operate at the lowest level of chromatin organization, i.e., by activating the chemical modifications of genomic loci and/or of histone tails. Post-translational modifications of histone tails can in turn generate docking sites or modulate the affinity of nuclear proteins for chromatin [6]. Crucially, many chromatin remodelers operate at the nano-/mesoscale, from a few nanometers up to 200–300 nm. In the context of high-order chromatin organization, a pivotal role is played by the polycomb group proteins (PcGs) [7]. PcGs constitute a large family of proteins which assemble into multiprotein complexes that are able to establish a transcriptionally repressive chromatin state which changes over time by a synergistic combination of chemical histone modifications with physical crosslinking of distal genomic regions [7]. The PcG machinery is present throughout most eukaryotic lineages and is known to play a key role in embryonic stem cell biology [7] and cell lineage commitment [8], as well as being involved in malignant transformation processes [9].

Knowledge of multiscale genome organization and multifaceted gene regulation is still in its infancy. However, techniques for mapping genomic regions with increasing spatial resolution have made considerable progress in the last two decades. The dawn of this new era was marked by the appearance of new techniques mapping genome organization by ligating linearly distal regions that come into 3D spatial proximity [5]. In addition, a remarkable contribution was allowed by the recent developments of fluorescence super-resolution microscopy (SRM), a toolbox of imaging techniques highly suited to address the meso-/nanoscale of TAD and CND organization since they are able to break the classical diffraction limit of conventional optical (fluorescence) microscopes [10]. SRM leverages detection modalities which combine the ability to recognize single emitters and the shaping of the illumination beam, enabling the visualization of structures only a few tens of nanometers apart by separating them in time and space, respectively [11]. Some SRM approaches have—in principle—no spatial resolution limit, and imaging at the molecular scale has been repeatedly demonstrated [12]. Practically, SRMs seldom achieve the routine <10 nm resolution of electron microscopy, which, since the seminal studies of Bernhard et al. sixty years ago [13], has strongly contributed to the understanding of chromatin structure in situ [14,15]. Compared to electron microscopy, however, SRMs enable the recognition of several molecular signatures at a time (multiplex functional imaging) and the dynamic changes occurring in living cells to be addressed.

The purpose of this paper is to provide an updated review of SRM studies aimed at PcG regulation of chromatin epigenetic states. Accordingly, we will briefly review the basic idea and the major features of SRM (Section 2). Then, we will describe the nature of polycomb proteins and their organization in multiprotein complexes (Section 3 and Section 4). Finally, we will review the SRM studies carried out to understand how PcGs modulate chromatin topology (Section 5) and X-chromosome inactivation (Section 6). A concluding paragraph will recapitulate the present knowledge on this topic.

## 2. Super-Resolution Microscopy (SRM)

In this section, we provide a short introduction to the basic idea of SRM and the four main families in which it is currently articulated. The reader is referred to more specialized reviews for comprehensive descriptions of this field of microscopy [10,11,12].

From a purely physical perspective, the light emitted by any point source—for instance, a fluorescent molecule in the focus of a microscope objective—undergoes diffraction, and the point will appear in the image as the so-called Airy diffraction pattern [16]. Owing to the reversibility of optical pathways, the same phenomenon occurs when light is focused at one point, for example, when a sample is illuminated to excite fluorescence. The 3D region individuated by the Airy diffraction pattern is called the point spread function (PSF). The finite size of the PSF limits the spatial resolution of the optical system, according to an equation developed by the physicist Ernst Abbe, which states that the minimal distance *d* at which two signals may be distinguished by a microscope (actually, the highest spatial frequency of the image) is given by [17]:

d=λ/2NA

where *λ* is the wavelength of the probing radiation and *NA* is the numerical aperture of the microscope objective. In practice, popular confocal microscopes are limited to approximately 200–300 nm of lateral resolution (in the xy-plane of the sample) and 600–1000 nm of axial resolution (along the z-axis of the sample). Nonetheless, this limit has been demonstrated to be apparent. Following the original idea of Toraldo di Francia, the resolving power of an optical instrument, such as a microscope, is not a well-defined physical quantity, as it depends critically on the amount of information about the object being observed [18]. For instance, the resolving power of a confocal microscope is limited by the fact that all fluorophores residing in the excitation PSF are quasi-simultaneously excited and emit light together. Therefore, their emissions diffract together and are detected together as the product of excitation and emission PSFs. In order to overcome this limitation, we must introduce additional information into the system. For example, we may alter in a controlled way the probability of the molecules emitting in the excitation region, separating their emissions in space and/or in time and preventing them from being detected together. This approach is the key to super-resolution microscopy.

**Figure 2:**
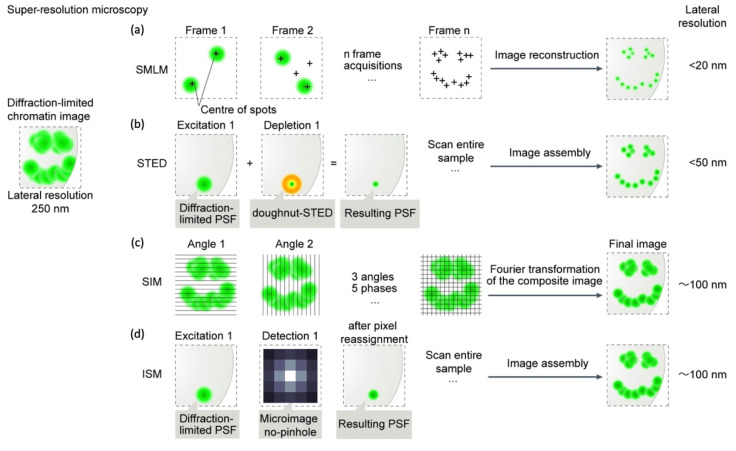
Super-resolution microscopy techniques. (**a**) Structured illumination microscopy (SIM). (**b**) Single molecule localization microscopy (SMLM). (**c**) Image scanning microscopy (ISM). (**d**) Stimulated emission depletion microscopy (STED).

The SRM family with the highest resolution improvements is called single molecule localization microscopy (SMLM; Figure 2a) [19]. SMLM includes stochastic optical reconstruction microscopy (STORM [20]), as well as its variant direct STORM (dSTORM [21]), and fluorescence photo-activated localization microscopy (F-PALM [22,23]). In SMLM, fluorophores belonging to the same excitation PSF are separated out in the temporal dimension by collecting their emissions one at a time. In practice, SMLM leverages fluorophores that can be converted from a fluorescent (or activated) state to a dark (or inactivated) state, either irreversibly or reversibly. The excitation mode ensures a stochastic emission of only a few fluorophores at a time, ensuring that individual emitters do not overlap their PSFs in a single imaging frame. The sample undergoes several activation/inactivation cycles, which result in thousands of acquisition frames, each characterized by a few, spatially sparse, emitting single molecules. The precise localization of the center of each single molecule is then retrieved by a post-acquisition algorithm, generating super-resolution images with a lateral resolution as low as 10–20 nm. Actually, in SMLM, resolution does not have its classical meaning, being replaced by the *localization precision* of the single molecules. Nonetheless, the astounding resolution of SMLM comes at the price of long acquisition times, since the localization precision is inversely correlated with the square root of the number of acquired photons [19]. This usually restricts STORM and F-PALM approaches to fixed samples, although living cells have been studied through the adoption of tailored strategies and/or fluorophores [24,25]. SMLM can also be performed in 3D mode [19]. Of note, STORM and dSTORM have been widely applied in super-resolution chromatin studies [4].

A second approach to SRM separates emitting molecules by means of excited state depletion by stimulated emission, which is triggered by strong laser illumination on the low-energy tail of the dye’s emission spectrum [26]. This approach, called stimulated emission depletion (STED; Figure 2b), leverages a confocal configuration supplied with a donut-shaped depletion laser beam superimposed upon the focused excitation spot, thereby keeping all fluorescent molecules dark except those at the center of the donut (Figure 2). Raster-scanning the sample allows the sequential registration of fluorescence from only those dyes that are effectively excited at each spatial location [26]. STED may reach a lateral resolution of 30–50 nm, albeit the strong laser intensity requires specific fluorophores [27]. Lower depletion intensities can be obtained by exploiting fluorophore lifetime [28] or through a modified version called reversible saturable optical fluorescence transitions (RESOLFT), which requires reversibly photoswitching fluorophores [29]. Like SMLM, STED has been repeatedly applied to investigate chromatin organization [30].

Both SMLM and STED do not have in principle theoretical resolution limits [12], because there is no theoretical (information) limit in separating out single emitters in the excitation PSF with infinite spatial precision. In practice, resolution limits are mostly related to the fluorescent labels and the technical implementation of SRM. Recently, a new approach called MINFLUX, which relies on the localization of individually switchable fluorophores with a probing donut-shaped excitation beam has been demonstrated to reach <10 nm resolution with minimum photon fluxes [31]. In MINFLUX, about two orders of magnitude fewer photons are required to achieve equivalent resolutions to the best-performing SMLM techniques [32]. However, MINFLUX instruments first appeared on the market only in late 2019, and the technique is not widely available.

While STED has been utilized to image live samples [33,34,35], it is usually applied to image fixed samples. This is mostly due to the complex genetic encoding of fluorophores which comply with the strong depletion intensities of STED (particularly in conventional continuous-wave STED) [27]. The need for specific fluorophores also reduces the spectral multiplexing of STED. Some of these issues are addressed, albeit at the cost of lower resolution gains (about twofold over diffraction-limited microscopy), by less challenging SRM techniques which leverage the structuring of the excitation pattern or of the detection device and offer an effective strategy to image living samples.

Structured illumination microscopy (SIM; Figure 2c) leverages a non-uniform illumination pattern whose interference with the spatial frequencies of the sample (the “Moirè effect”) generates an emission pattern that can be analyzed in the Fourier mathematical space to improve the resolution (Figure 2) [36]. A major advantage of SIM is its full compatibility with standard fluorophores and labelling procedures; it also enables multicolor imaging of living cells. SIM was applied early in chromatin studies [37] and has allowed the visualization of CNDs contained in TADs, demonstrating that ~100–120 nm resolution may be sufficient to visualize chromatin subdomains [38,39].

A more flexible alternative to SIM is represented by image scanning microscopy (ISM; Figure 2d) [40,41]. The optical sectioning ability of a confocal microscope is mainly due to the pinhole spatial filter placed in a conjugate image plane in front of the detector along the fluorescence detection path. Ideally, by closing the pinhole below 0.2 AU, *d_xy_* would be improved by a factor $\sqrt{2}$ compared to 1 AU, at the price of a dramatic decrease in the signal reaching the detector (95%) and severe degradation of the signal-to-noise ratio (SNR) of the final image. ISM overcomes this limitation by replacing the single point detector of a confocal microscope with a detector array [42,43]. Each element of the array generates a “confocal” image of the sample, the images differing in terms of information content, as they map different spatial regions of the point spread function generated by the light coming from the objective focus. By the “pixel-reassignment method”, i.e., the sum of all the scanned images after a spatial shift and intensity normalization, an ISM image (PR-ISM) is generated with an effective radial resolution improvement ≥$\sqrt{2}$, with an SNR equivalent or larger than that obtained at 1 AU (Figure 2) [44]. To extend the resolution further down to 100–120 nm, ISM usually makes use of an adaptive multi-image deconvolution algorithm. Despite these advantages, ISM has only been minimally applied to investigate the organization of chromatin at sub-diffraction resolution [4], despite its robust and versatile technical implementation, which affords high spectral multiplexing and may also be compatible with lifetime detection, affording further layers of imaging contrast [40], although a recent paper by the present authors applied ISM to investigate the functional colocalization of PRC1 and PRC2 in differentiated lung cells [45].

## 3. Structure of Polycomb Repressor Complexes PRC1 and PRC2

**Figure 3:**
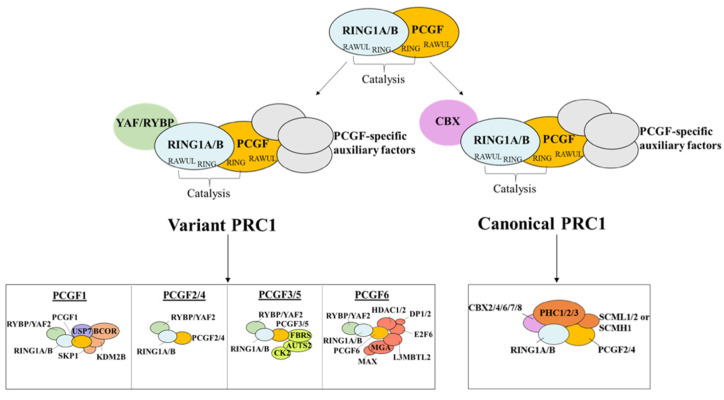
Structure of the polycomb repressor complex 1 (PRC1). PRC1 subdivides into “variant” (vPRC1) and “canonical” (cPRC1) complexes. Really Interesting New Gene 1 A/B (RING1A/B) is common to both. vPRC1 assembles around one of six Polycomb Group RING Finger (PGCF) proteins (PCGF1–PCGF6), whereas cPRC1 contains only PCGF2/4. Further components, such as YY1-Binding Protein (RYBP) and ChromoBoX proteins CBX2/4/6/7/8, are contained only in vPRC1 and cPRC1, respectively. For further description, see text.

**Figure 4:**
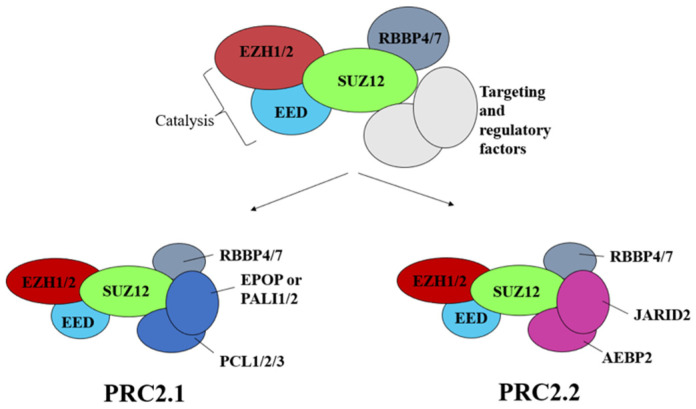
Structure of the polycomb repressor complex 2 (PRC2). The core of PRC2 is composed of EZH1/2, Embryonic Ectoderm Development (EED), Supressor Of Zeste 12 Homolog Protein (SUZ12), and Retinoblastoma Binding Protein 4 or 7 (RBB4/7). Further subdivision in PRC2.1 and PRC2.2 is given by additional proteins, among which Jumonji And (A+T)-RIch Interaction Domain-containing protein 2 (JARID2) and Adipocyte Enhancer-Binding Protein 2 (AEBP2) play crucial roles in the initiation of PRC1/2 repressing activity (see text).

Originally, the name “polycomb” was used for a *Drosophila* mutant showing improper body segmentation on account of misregulation of the *Hox* genes [46]. Polycomb Group proteins (PcG) have since been identified as a group of negative transcription factors sustaining the formation of facultative heterochromatin and thereby dynamically defining key aspects of cell identity and activity [7,47]. PcG proteins assemble into protein complexes that are highly conserved from flies to mammals [48] and work through the post-transcriptional modification of histones at two marks: trimethylation at lysine 27 (H3K27Me3) of Histone H3 and ubiquitination at lysine 119 of histone H2A (H2AK119Ub) [49]. Most PcG proteins assemble into two main protein complex groups, Polycomb-repressive complexes 1 (PRC1; Figure 3) and Polycomb-repressive complexes 2 (PRC2; Figure 4). PRC1 and PRC2 are responsible for the deposition of H2AK119Ub and H3K27me3, respectively [47].

In mammals, PRC1 is characterized by six different forms (PRC1.1-PRC1.6) [50], whereas PRC2 exists in two different main forms (PRC2.1 and PRC2.2) [7]. The core component of PRC1 is a heterodimer of one of the two E3-ubiquitin ligases RING1A/B (Really Interesting New Gene 1 A or its alternative isoform B) and one of the six PCGF1-6 (Polycomb Group Ring Finger) paralogs (Figure 3). In “variant” PRC1s (vPRC1), RING1A/B associates with either PCGF1, 3, 5, or 6 (Figure 3). vPRC1s also include YY1-binding protein (RYBP) or its paralogue YAF2, along with various additional subunits depending on the PCGF component. Of note, vPRC1s are responsible for most H2AK119Ub deposition, given their enhanced E3-ligase activity due to RYBP or YAF2 [51]. The core of “canonical” PRC1 (cPRC1) is the heterodimer of RING1A/B with either PCGF2 or 4 (Figure 3). cPRC1s also includes one of the CBX2/4/6/7/8 (Chromobox Homologs) proteins, one of the PHC1-3 (Polyhomeotic Homolog) proteins, and SCMH1/L2 (Sex Combs on Midleg Homolog 1/Like 2). CBX proteins are characterized by chromodomains that can bind to H3K27me3 and/or H3K9me3 repressive marks and are thought to play a key role in recruiting PRC1 at PcG-repressed sites [52]. PHC1-3 proteins contain a Sterile Alpha Motif (SAM) domain that allows the formation of long-range interactions among PRC1 complexes [53]. cPRC1s displays much lower ubiquitination activity compared to vPRC1 [54], but it is tailored to alter chromatin structure and topology [53,55].

PRC2 complexes assemble around a tetrameric core composed of EZH2/1 (Enhancer of Zeste Homolog 2 or its paralog 1), EED (Embryonic Ectoderm Development), SUZ12 (Supressor Of Zeste 12 Homolog Protein), and RBBP4/7 (Retinoblastoma Binding Protein 4 or 7) (Figure 4) [7]. The histone methyltransferase (HMTase) activity of PRC2 is conferred by EZH2/1, and it is enhanced by a positive feedback loop relying on the specific recognition of the H3K27me3 by EED, followed by allosteric activation of PRC2 by the same protein [56]. PRC2 splits further into two sub-complexes, PRC2.1 and PRC2.2 (Figure 4), whose different protein components aggregated to the tetrameric core modulate HMTase activity and/or its targeting to DNA (Figure 4) [57,58].

## 4. Formation and Spread of Polycomb Domains onto Chromatin

**Figure 5:**
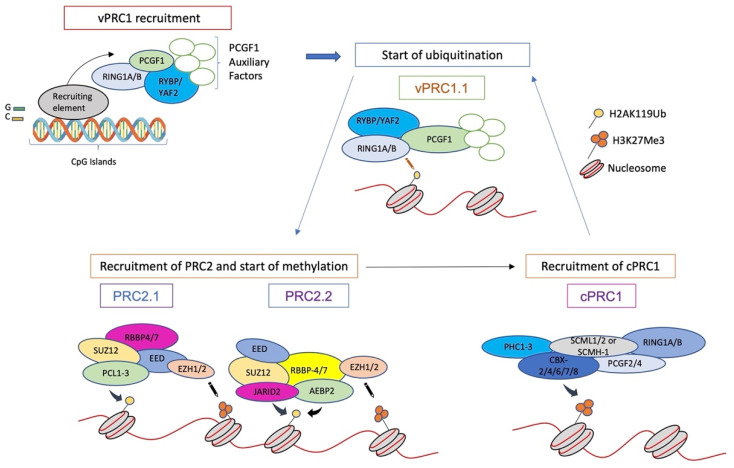
The “reverse hierarchical” model of PRC1 and PRC2 activity. For description, see text and ref. [7].

The first model of PcG recruitment onto chromatin was inferred from *Drosophila* studies and describes a hierarchical interplay between the polycomb complexes, where PRC2 is recruited first at Polycomb Response Elements (PREs) by sequence-specific DNA-binding factors, methylation of H3 occurs, and this further recruits PRC1 [59]. This very simple model has been more recently questioned by a study that showed no unique hierarchy for coordinated recruitment of PRC1 and PRC2 at polycomb response elements [60]. In mammals, PcGs mainly identify target gene promoters and other regulatory elements through their association with unmethylated CpG islands (CGIs) [61]. CGIs are short (1–2 kb) regions of CpG-rich DNA that are associated with approximately 70% of mammalian gene promoters [62]. The lysine-specific demethylase 2B (KDM2B), which participates in vPRC1.1, contains a zinc-finger-CXXC domain that specifically binds to unmethylated CpGs, thereby docking vPRC1.1 to CGIs in embryonic stem cells (ESCs) [63,64]. Thus, a “reverse hierarchical” mechanism of PcG action has been proposed in recent times [47]. In this model (Figure 5), the first step is the recruitment of vPRC1.1 onto chromatin through KDM2B and the subsequent deposition of the H2AK119Ub mark [63,65]. Then, histone modification is recognized by the AEBP2 and JARID2 modules of PRC2.2 [65,66]. PRC2 recruitment at unmethylated CGIs is further supported by PCL proteins in PRC2.1 [67,68]. Recruitment of PRC2 activates the deposition of H3K27Me3 [69], to which cPRC1 eventually docks through its CBX modules [52,70]. Interestingly, from their initial recruitment foci, PcG proteins seem to be able to spread their repressive marks bidirectionally, generating a repressive landscape for a target genomic region [7,71]. This can be ascribed to the two positive recognition/histone modification feedback mechanisms prompted by EED [72] and RYBP [73] in PRC2 and PRC1, respectively. These mechanisms also should account for the propagation and replenishment of these histone marks during DNA replication, thereby sustaining memory of polycomb repression upon cell cycle and division [73,74].

Although the actual mechanism of transcription repression by chromatin-bound PRC1/PRC2 is still partially obscure, at least two main processes have been identified [53,75]. At the nanoscale (~10 nm, 1–10 kb), CBX2 can locally force adjacent nucleosomes into compact clutches through liquid–liquid phase separation (LLPS) driven by its charged structure and the presence of an intrinsically disordered region (IDR) [76,77,78]. At the mesoscale (~100 nm, 10–100 kb), the SAM domain of the PHC module of cPRC1 enables the compaction of distal chromatin regions, generating long-range repressive chromatin multi-looped structures [79,80,81]. In both cases, chromatin compaction is believed to make the underlying genomic region inaccessible to the transcription machinery, although PcG features distinct from cPRC1 should contribute to the transcription repression [7]. Of note, the mesoscale compaction of chromatin by polyhomeotic proteins has been the only topic addressed by SRM in the context of PcG-mediated transcription repression and will be reviewed in the next section.

## 5. The Role of PRC1 in Shaping Chromatin Topology

**Figure 6:**
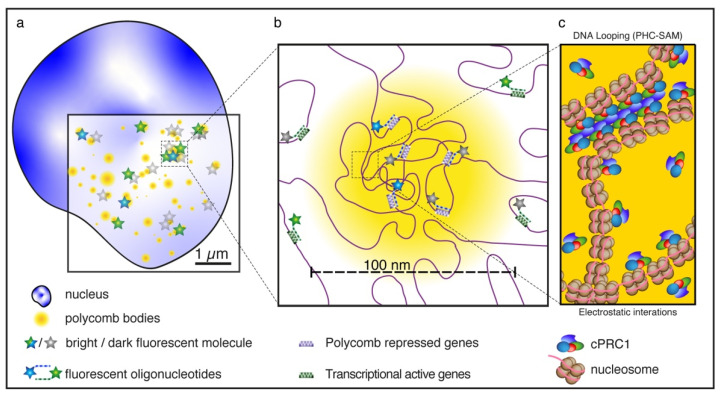
Scheme of 3D STORM studies on polycomb nanodomains in *Drosophila*, after ref. [85,86]. Polycomb-controlled genes, such as the *Hox* genes, are labelled by oligonucleotides bearing blinking fluorophores for STORM imaging (**a**), thereby revealing compacted chromatin regions ~100 nm in size (**b**). The compact chromatin state of polycomb-repressed domains could be directly linked to the multimerization ability of the polyhomeotic component of PRC1 through the Sterile Alpha Motifs (SAMs) they embed (**c**).

The assembly of polycomb proteins into nuclear foci in the cells of flies and mammals, supposedly to bring PcG-regulated genes together, has been revealed by diffraction-limited fluorescence microscopy in the nineties [82,83,84]. However, only in 2016 were the first two papers reporting on the use of SRM to investigate the role of PcG in chromatin topology published [85,86]. Both these seminal studies leveraged 3D STORM [87] to examine the PcG-driven chromatin assembly in *Drosophila* cells. Figure 6 provides a graphical description of the main aspects of these studies.

In the first study, Boettiger et al. [85] addressed a compelling question: how to reconcile the hierarchical organization of chromatin inferred from chromatin conformation capture measurements [88] with the functional demarcation of chromatin in domains of distinct epigenetic states characterized by biochemical modifications and DNA-binding proteins [89]? In simpler terms: how does chromatin organize in 3D in different epigenetic domains? For this challenge, Boettiger combined 3D STORM with the novel “oligopaint” fluorescence in situ hybridization (FISH) approach [90] to reveal the chromatin ultrastructures (20 nm *xy*- and 50 nm *z*-resolution) of three epigenetically distinguishable sub-Mb domains of the *Drosophila* genome: (1) transcriptionally active, (2) polycomb-repressed, and (3) transcriptionally inactive domains [85]. Of note, these three domains had been previously stratified from ChIP-seq (chromatin immunoprecipitation followed by sequencing) and DamID (DNA adenine methyltransferase identification) data on the basis of the enrichment of histone modifications and regulatory proteins, such as PcGs. Three-dimensional STORM highlighted the volumes of hybridization regions and showed a power-law scaling dependence on genomic length, albeit with different exponents. Active regions showed the lowest chromatin packing densities and a superlinear dependence (scaling exponent > 1) on genome length. Conversely, the highest compaction was observed for the polycomb domains, for which a scaling exponent <1 indicated that the packaging density increases with domain length. These data could be easily reconciled with previous FISH studies using conventional imaging in mammalian cells [91,92]. However, 3D STORM, also in two-color mode, revealed intriguing additional features of polycomb-repressed domains, namely, their high degree of chromatin intermixing across the domain and their ability to spatially exclude neighboring active regions. This contrasted with active and inactive domains, which were found to be partially intermixed with each other and for which small subdivisions were found to possess similar scaling behaviors to their host domains. Boettiger et al. [85] elegantly demonstrated by knockdown experiments that the observed features of the compact chromatin states of polycomb-repressed domains could be directly linked to the polyhomeotic (Ph—the *Drosophila* analog of PHC1-3 in mammals) multimerization ability and not to the catalytic activity of *Drosophila* PRC1. Of note, modeling experiments suggested that the self-interacting properties of the SAM domain of Ph could be the basis of the observed biophysical differences between PcG-repressed and active/inactive regions.

The latter hypothesis was fully demonstrated in the second seminal paper of 2016 [86]. By single molecule localization (STORM mode) of target proteins immunolabeled with fluorescent antibodies, Wani et al. [86] showed that Pc and Ph components of *Drosophila* PRC1 assemble into numerous (600–800) aggregates of 110–140 nm in the cell nucleus, hinting at the pervasive nuclear clustering of PRC1. Strikingly, the mutation of one of the two polymerization interfaces of the SAM motif (Ph-ML) fully disassembled the PRC1 clusters, demonstrating that multi-scale clustering of Ph depends on the oligomerization capacity of its SAM domain. Monitoring chromatin topology by 4C-seq in tandem with cluster manipulation revealed that SAM-driven aggregation of PRC1 facilitates long-range chromatin interactions, particularly for distal sequences separated by >2 Mb. Removal of these interactions by abolishing SAM-driven clusterization resulted in observable changes in gene expression, including the de-repression of some well-known PcG target genes and changes in the 3D organization of chromatin fibers [47]. The comparison of molecular simulations with cluster size vs. Ph localization inferred by STORM hinted at a “bridging model”, where hundreds of molecules or primary clusters bound at a cPRC1 node polymerize with those bound at other, non-adjacent nodes on the chromatin polymer, forming a long-range network of chromatin contacts. The introduction of SAM-defective Ph molecules “caps” primary clusters at nodes, thereby avoiding their spreading to distal chromatin regions [86].

Just one year later, the crucial role of SAM-driven PRC1 clusterization in the topological organization of mammalian chromatin was visually demonstrated by an elegant study of Kundu et al. leveraging 3D STORM spatial resolution [80]. These authors investigated the extended network of cPRC1-driven chromatin interactions framing the repressed *Hox* genes in mouse embryonic stem cells [93], which had been previously studied by diffraction-limited FISH imaging [94]. Consistently with 5C chromosome conformation capture data, FISH by 3D STORM identified discrete and compact 20 to 140 kb PRC1 domains at *Hox* and other developmental loci in mouse ESCs and neural progenitors (NPCs) which were disrupted upon knockdown of the *Phc1* gene [80]. Besides being much shorter than TADs, these PRC1 domains were found to span across TAD borders, suggesting that chromatin folding allows for different hierarchies through the action of different factors. Kundu et al. [80] also showed that Variant PRC1 complexes and H2A ubiquitylation are neither necessary nor sufficient for the formation of these domains. Of note, Isono et al., via biochemical and diffraction-limited FISH/immunofluorescence imaging, had previously demonstrated the essential role of the SAM domain of mouse polyhomeotic PHC2 for cPRC1 clustering to sustain the stable target binding of PRC1/PRC2 and the robust gene silencing activity of the PcG machinery [79]. Taken together, these data support a model conserved from *Drosophila* to mammals: the SAM motif of polyhomeotic isoforms enables PRC1 (cPRC1 in mammals) to reorganize the chromatin such that the bound loci become isolated from the surrounding non-PRC1-bound chromatin and interact primarily with other bound loci by looping out the intervening non-PcG-bound chromatin.

A more recent work [95] questioned the apparent consistency of the chromosome capture and STORM data collected by Boettiger et al. [85] to provide a realistic picture of PRC1-driven chromatin compaction in *Drosophila*. Indeed, Liu et al. applied a polymer-based chromosome modeling approach, termed the heterogeneous loop model (HLM) [96], to chromosome capture and STORM data, and found them to be poorly consistent with each other [95]. Of note, the same authors highlighted that previous works addressing *Drosophila* chromatin organization by FISH in STORM mode, albeit not directly focused on PcGs, clearly showed that PcG-repressed domains framed compaction states of chromatin similar to inactive domains [39,97]. These studies paralleled measurements by King et al. [51] that revealed reduced chromatin accessibility even by knocking out RING1b or EED. Liu et al. pointed out that this discrepancy cannot be explained solely by the so-called “FISH-HiC paradox” [98], i.e., the significant differences between the cell ensembles considered by the two techniques (i.e., millions of cells in Hi-C and a few tens in FISH). However, in their opinion, the cell model and the fixation procedure (much harsher in FISH) could have effectively biased Boettiger’s measurements [85]. A further source of bias in the assessment of chromatin compaction could be linked to the extensive pairing of homologous chromosomes that occurs in *Drosophila*, which leads to juxtaposed, albeit physically distinct, TADs, as revealed by SIM [39]. The “FISH-HiC paradox” itself has been questioned by studies that have revealed a very nice correlation between HiC and FISH imaging in SIM mode [39,88], supporting the idea that SRMs are able to recapitulate chromatin topology at the nanoscale in both *Drosophila* and mammals. Overall, this debate hints at the complex issues that must be carefully addressed in all cutting-edge studies targeting biological spatial scales unexplored by SRMs.

## 6. Polycomb Complexes and Xist Regulation

PRC1 and PRC2 play crucial roles in the context of X-chromosome inactivation (XCI), i.e., the lifelong silencing of one X chromosome (Xi) in mammalian females to balance gene dosage between sexes [99]. The current model of XCI involves the repressive “coating” of the cis region of Xi by the long noncoding RNA *Xist*, which is transcribed from Xi. XCI starts early in embryogenesis and is initiated by *Xist*-assisted recruitment of repressive complexes, such as SPEN, vPRC1/PRC2, and other repressive or structural proteins, to nucleation sites located all along the Xi [100,101]. At the end of the process, about 1000 genes spanning over 167 Mb are silenced [102]. Noticeably, silenced Xi regions are enriched in H3K27Me3 [103] and H2AK119ub marks [101], supporting the crucial role of PcG complexes in this process [101,104]. Epigenomic methods based on ensembles of millions of cells have led to a model in which *Xist* and its interactors form ribonucleoprotein complexes broadly distributed along Xi [103]. This model was supported by conventional diffraction-limited fluorescence microscopy studies [105]. However, the situation has radically changed with the advent of super-resolution microscopy [102,106,107,108].

Most notably, in 2014, Smeets et al. revealed in a 3D SIM FISH/immunofluorescence study that only 50–100 *Xist* molecules decorated mammalian Xi, creating distinct foci, and this number was paralleled by EZH2 foci [107]. These results were then confirmed by a subsequent paper using a 3D-STORM approach by Sanwoo et al. [108]. Overall, these data suggested that the number of *Xist* molecules on the Xi was much lower than previously thought [109]. Very consistently, ~100 *Xist*-containing complexes at the docking site of Xi, each embedding about two *Xist* molecules, have been recently quantified by an elegant SIM multicolor approach by Markaki et al. [102]. However, Markaki et al. [102] and Sanwoo et al. [108] differed—significantly—over the proposed mechanisms of large-scale Xi silencing. From the stoichiometry of *Xist* complexes and the large number of silenced genes on the X chromosome, Sunwoo et al. [108] hypothesized a “hit-and-run” model of *Xist* complexes shutting off genes while moving along Xi. This model was, however, eventually ruled out by the single particle tracking measurements of Markaki et al., which showed the sharp confinement of *Xist* complexes in well-defined genomic loci proximal to the *Xist* locus and enriched for repressive complexes [102]. Additionally, 3D SIM imaging revealed that *Xist* nucleates nanoscale supramolecular complexes (SMACs), which include hundreds of silencing proteins that accumulate into discrete Xist foci [102,110,111,112]. SMACs are dynamic entities which generate local protein gradients that are able to regulate proximal chromatin regions. The recruitment of vPRC1 (containing PCGF3/5) to *Xist* foci, mediated by the hnRNP K protein, activates the usual cascade of events leading to histone decorations and PRC2/cPRC1 multiscale assembly (Figure 5) [113]. From this evidence and the recently stratified knowledge about the topological modulation of chromatin by PcGs, Markaki et al. hypothesized that PcG deposition via SMACs may also induce chromatin compaction and the increase in SMAC densities around distal genes, which would explain how silencing propagates across the X chromosome. Genes in close proximity to the Xist phase-separated domains are silenced early, while more distal genes are silenced once the Xi becomes compacted through the action of PRC1 [102,112]. This mechanism is consistent with the non-dispensable role of PRC1/PRC2 in *Xist*-mediated gene silencing [101,113], in line with the suggested role for PcGs as “silencing stabilizers” of already silenced genes rather than active abrogators of gene transcription [114]. This model also coheres well with the poor colocalization of *Xist* RNA with PRC1/2 complexes and H3K27me3 that was revealed early on by the 3D SIM study of Cerase et al. [106]. It is possible that the recruitment of PRC2 into SMACs leads to a larger and time-dependent spacing of EZH2 from *Xist* by means of phase separation [115], as PRC2 does not seem to be directly recruited by *Xist* RNA [101,106].

In the context of STORM studies applied to PcGs, it is worth noting that both the effect of multiple blinking of fluorophores on stoichiometry and the nanoscale fluorophore spacing due to sequence localization plus the presence of antibody–antibody complexes were carefully evaluated by means of ingenious engineered cellular models in the study of Sunwoo et al., resulting in a resolution of >30 nm (localization precision: 20 nm) [108]. The effect of antibody–antibody spacing had been previously investigated also in the 3D SIM study of Smeets et al., resulting in a resolution of about 100 nm [107], in line with the lower resolution of 3D SIM as compared to STORM.

## 7. Conclusions

Super-resolution fluorescence microscopy approaches (SRMs) have revolutionized the field of biological imaging. The possibility of inferring the functional properties of biomolecules below the classical diffraction limit (200–250 nm) enables the understanding of a crucial hallmark of cellular life, the widespread ordering of many biological processes in meso-/nanoscopic domains. Recently, this approach has been applied to visualize chromatin topology. Indeed, the idea of the nucleus as a highly organized organelle has existed for over a century. Still, only the almost concomitant application of chromosome conformation capture methods and super-resolution microscopy has started to unveil the intricate networks of interchromatin interactions that modulate the flow of information from the genome and ultimately the phenotype of the cell. Polycomb group proteins (PcGs) are among the most-studied transcription factors, as their assembly in multifaceted complexes allows the dynamic local repression of gene transcription. In this paper, we reviewed the current knowledge of polycomb activity as inferred by super-resolution studies. Notably, the attention of researchers has mostly been focused on polycomb-dependent intrinsic chromatin properties (e.g., local compaction of polycomb-regulated domains), as revealed by fluorescence in situ hybridization (FISH) of target genomic loci carried out by single molecule localization in STORM/dSTORM mode. Super-resolution has contributed much less to present knowledge of how PcG assembly directs its activity, which nonetheless has been inferred in several genomic ensemble studies and a few cases of conventional diffraction-limited imaging research. This knowledge has been condensed into a complex working model which hints at the superbly coordinated activity of this family of proteins at the mesoscale. Overall, SRM provides a new window to observe 3D chromatin topology modulated by PcGs.

## Data Availability

Data contained within the article are available on request from the authors.
